# Psychosocial stressors and current e-cigarette use in the youth risk behavior survey

**DOI:** 10.1186/s12889-023-16031-w

**Published:** 2023-06-06

**Authors:** John Erhabor, Ellen Boakye, Ngozi Osuji, Olufunmilayo Obisesan, Albert D. Osei, Hassan Mirbolouk, Andrew C. Stokes, Omar Dzaye, Omar El-Shahawy, Carlos J. Rodriguez, Glenn A. Hirsch, Emelia J. Benjamin, Andrew P. DeFilippis, Rose Marie Robertson, Aruni Bhatnagar, Michael J. Blaha

**Affiliations:** 1 Ciccarone Center for Prevention of Cardiovascular Disease, Johns Hopkins, 600 N Wolfe St, Blalock 524, Baltimore, MD 21287 USA; 2 The American Heart Association Tobacco Regulation and Addiction Center, Dallas, TX USA; 3 Department of Medicine, MedStar Union Memorial Hospital, Baltimore, MD USA; 4 Department of Internal Medicine, Yale School of Medicine, New Haven, CT USA; 5 Department of Global Health, Boston University School of Public Health, Boston, MA USA; 6 Department of Population Health, New York University School of Medicine, New York, NY USA; 7 Albert Einstein College of Medicine, Bronx, New York, NY USA; 8 Division of Cardiology, Department of Medicine, National Jewish Health, Denver, CO USA; 9 Cardiovascular Medicine, Boston Medical Center, Boston University School of Medicine, Boston, MA USA; 10 Department of Epidemiology, Boston University School of Public Health, Boston, MA USA; 11 Department of Medicine, Vanderbilt University Medical Center, Nashville, TN USA; 12 University of Louisville School of Medicine, Louisville, KY USA

**Keywords:** E-cigarette use, Tobacco use, Adolescents, Youth, Stressors, Psychosocial stressors

## Abstract

**Background:**

This study explores the association between psychosocial stressors and current e-cigarette use among adolescents in the United States.

**Methods:**

We used data from 12,767 participants in the 2019 National Youth Risk Behavioral Survey to examine the association between psychosocial stressors (bullying, sexual assault, safety-related absence from school, depressive symptoms, suicidal ideation, physical altercation, and weapon threats) and past-30-day e-cigarette use using multivariable-adjusted logistic regression models. We examined the association for each stressor and then as a burden score (0–7). To compare the strength of the association between stressors and current e-cigarette use to current combustible cigarette use, we additionally examined the association between each stressor and current combustible cigarette use.

**Results:**

Approximately 32.7% reported current e-cigarette use. The weighted prevalence of current e-cigarette use was higher among individuals who experienced stressors than those who did not. For example, bullying (43.9% vs. 29.0%). Similar prevalence patterns were seen among other stressors. Individuals who experienced stressors had significantly higher adjusted odds of current e-cigarette use than those who did not (OR [Odds Ratio] range: 1.47–1.75). Similarly, individuals with higher burden scores had a higher prevalence (zero [20.5%], one [32.8%], two [41.4%], three [49.6%], four to seven [60.9%]) and higher odds of current e-cigarette use (OR range: 1.43–2.73) than those with a score of zero. The strength of the association between the stressors and e-cigarette use was similar to that between the stressors and combustible cigarette use.

**Conclusion:**

The study demonstrates a significant association between psychosocial stressors and adolescent e-cigarette use, highlighting the potential importance of interventions, such as targeted school-based programs that address stressors and promote stress management, as possible means of reducing adolescent e-cigarette use. Future research directions include exploring underlying mechanisms linking stressors to e-cigarette use and evaluating the effectiveness of interventions addressing stressors in reducing adolescent e-cigarette use.

**Supplementary Information:**

The online version contains supplementary material available at 10.1186/s12889-023-16031-w.

## Background

As defined by the World Health Organization, adolescence encompasses the phase of life between childhood and adulthood, from 10 to 19 years [1]. Tobacco consumption frequently commences and becomes established during this developmental period, with approximately 90% of adults who use combustible cigarette in the United States (U.S.) initiating tobacco use by age 18, and 98% by 26 years [2, 3]. Given this early onset of tobacco use, it is not surprising that cigarette smoking currently persists as a primary preventable cause of disease, disability, and mortality globally [1, 4]. Despite efforts to reduce tobacco use, the World Health Organization reports that tobacco use kills more than 8 million people yearly and causes various health problems, including cancer, cardiovascular disease, and respiratory diseases [1].

Recent data from the Youth Risk Behavior Survey (YRBS) show a decline in current cigarette smoking, cigar smoking, and smokeless tobacco use among adolescents [5]. This is a promising trend; however, the introduction of e-cigarettes and the ease at which these devices can be purchased have raised concerns about a possible reversal of years of progressive decline in tobacco consumption [6, 7]. The recently published Morbidity and Mortality Weekly Report, over two million middle and high school students reported current (past 30-day) e-cigarette use, with two in five reporting frequent e-cigarette use, and one in four reporting daily use [8]. E-cigarettes are handheld devices containing aerosols with nicotine, additives, aldehydes, formaldehyde, and other harmful or potentially harmful chemicals [9]. Nicotine in e-cigarettes can cause addiction and harm the developing brain [10, 11]. Although evidence varies, some studies suggest that e-cigarettes may act as a gateway to using other tobacco products like combustible cigarettes [12, 13]. Given these findings, ongoing monitoring of adolescent tobacco use and comprehensive strategies aimed at addressing traditional and emerging tobacco product use among youth are essential.

Psychosocial stressors encompass a wide range of short and long-term adverse life events that can impact an individual's psychological well-being and social functioning, such as experiences of trauma, loss, interpersonal conflicts, financial difficulties, academic pressures, and exposure to discrimination or marginalization [14, 15]. Such stressors can prompt the initiation and continued use of tobacco products, as has been shown among middle-aged adults [16, 17]. Prior studies have also shown that psychosocial stressors such as depression are associated with youth cigarette smoking as some youth may use smoking as self-medication for depression and anxiety [18, 19]. Psychosocial stressors may therefore influence e-cigarette use—the most common tobacco product among youth. Among youth, such stressor may include bullying, fights, and depression. A 2020 study showed that bullying was significantly associated with e-cigarette use among sexual minority youth in the U.S [20]. Furthermore, a longitudinal study conducted in 2018 demonstrated that externalizing symptoms, encompassing conduct disorder, attention-deficit/hyperactivity disorder, and oppositional defiant disorder, as well as internalizing symptoms, such as anxiety and depression, were strong predictors of nearly all forms of tobacco use, including e-cigarette consumption, in both youth and young adult populations [21].

Prior research into the association between psychosocial stressors and adolescent e-cigarette use has been constrained by a narrow focus on specific stressors and reliance on convenience samples, which may limit generalizability. Our study addresses these limitations by exploring a broader array of stressors, utilizing a nationally representative dataset, and evaluating the cumulative impact of stressors on e-cigarette use. We employed the 2019 National YRBS, which is representative of high school students in the U.S., to examine the associations and cumulative burden of diverse stressors on adolescent e-cigarette use. We hypothesized that adolescents exposed to psychosocial stressors will exhibit a higher prevalence and increased likelihood of e-cigarette use compared to their unexposed peers. The results of this study could inform targeted interventions and policies to reduce e-cigarette use among adolescents experiencing psychosocial stressors.

## Methods

We used data from the 2019 YRBS, a cross-sectional nationally representative sample of U.S. high school students. The YRBS follows a three-stage cluster sampling design to generate a representative sample of students in grades 9 through 12. In 2019, the survey was administered in 78 locations across the U.S., including national and state levels, local school districts, territories, and tribal governments. A detailed description of the methodology used in the YRBS has been previously published [22].

Out of the 13,677 students who participated in the YRBS, we included only those who provided complete information on e-cigarette use, resulting in a final sample size of 12,797. The YRBS collects information every two years on health-risk behaviors that contribute to the leading causes of death and disability in students, including behaviors related to unintentional injuries and violence, substance use, unhealthy dietary habits, and insufficient physical activity. The school-level response rate for 2019 was 75.1%, while the student-level response rate was 80.3%, resulting in an overall response rate of 60.3% (i.e., [student response rate] × [school response rate]) [23]. Since we used publicly available, de-identified data, our study was exempt from institutional review board assessment.

### Assessment of psychosocial stressors

This study examined seven stressors, including bullying, sexual assault, school-related absence from school, depressive symptoms, suicidal ideation, physical altercations, and weapon threats. The seven stressors were selected based on relevant literature as well as the intra- and inter-personal components of the Social-Ecological Model, which posits five levels of factors that influence an individual’s behaviors (intrapersonal, interpersonal, institutional, community, and public policy) [24]. The specific questions used to assess each stressor have been presented in Supplementary Table 1.

### Assessment of e-cigarette and combustible cigarette use

E-cigarette use was assessed with the question: “During the past 30 days, on how many days did you use an electronic vapor product?” and combustible cigarette use was assessed with the question: “﻿During the past 30 days, on how many days did you smoke cigarettes?” Participants who reported using e-cigarettes in the preceding 30 days of the survey were classified as currently using e-cigarettes. Similarly, participants who reported smoking combustible cigarettes at least one day within the past 30 days were regarded as currently using combustible cigarettes.

### Other covariate

The sociodemographic variables included in this study were age, sex (female; male), race/ethnicity (American Indian/Alaskan Native/Native Hawaiian/Pacific Islander; Asian; African American; White; Hispanic; Multi-racial), grade level (9th, 10th, 11th, and 12th), and sexual orientation (Heterosexual; Gay or Lesbian; Bisexual; not sure). To assess other variables, participants were asked the following questions: "During the past 30 days, on how many days did you smoke cigarettes?" to assess combustible cigarette use; "During the past 30 days, on how many days did you use chewing tobacco, snuff, dip, snus, or dissolvable tobacco products, such as Copenhagen, Grizzly, Skoal, or Camel Snus?" to assess smokeless tobacco use; “﻿During the past 30 days, how many times did you use marijuana” to assess marijuana use; "During the past 30 days, on how many days did you smoke cigars, cigarillos, or little cigars?" to assess cigar use and "During the past 30 days, on how many days did you have at least one drink of alcohol?” to assess alcohol use. Participants who reported consumption of alcohol, marijuana, or any of the tobacco products within the preceding 30-day period, regardless of the frequency of use, were classified as currently using the respective substances.

### Statistical analysis

We categorized the study population into two groups: individuals who reported past-30-day use e-cigarettes and those who did not report such use, irrespective of other tobacco product use. Then, we summarized the demographic and tobacco use characteristics of the entire sample and for the two comparison groups using proportions. Thereafter, we estimated the weighted prevalence of current e-cigarette use by the seven unique psychosocial stressors. To test for correlation between psychosocial stressors we used the Pearson correlation test, which showed weak correlations for most of the stressors (0.11–0.30) and moderate correlation between depressive symptoms and suicidal thoughts (0.48).

We utilized logistic regression models with listwise deletion (complete case analysis) and adjustment for potential confounders, we examined the association between psychosocial stressors and current e-cigarette use. We sequentially adjusted for confounding variables using three models. In model 1, we adjusted for age, sex, race and ethnicity, sexual orientation, and body mass index. In model 2, we adjusted for the variables in model 1 and current use of combustible cigarettes, cigars, and smokeless tobacco, and in model 3, we adjusted for variables in model 2 and current marijuana and alcohol use.

To examine the association of the cumulative burden of psychosocial stressors with current e-cigarette use, we generated a composite psychosocial stressor burden score for each participant based on the number of stressors present. Since seven stressors were assessed, scores ranged from 0–7. The scores were categorized into four mutually exclusive groups: 0, 1–2, 3, and 4–7, to have adequate numbers in each category. We estimated the weighted prevalence of current e-cigarette use across the burden scores overall and by sex. Then, using multivariable logistic regression models with sequential adjustment for confounders as described above, we explored the association between the burden scores (zero being the reference group) and current e-cigarette use. We tested for the linear trend of the association between the psychosocial stressor burden score and current e-cigarette use using the post-estimation “*contrast”* command.

In the supplementary analysis, we restricted our sample to individuals who did not use any of the other tobacco products and then assessed the association between stressors and sole e-cigarette use. Finally, to compare the strength of the association between stressors and e-cigarette use to that of combustible cigarette use, we examined the association between each of the seven stressors and current combustible cigarette use, sequentially adjusting for confounding variables including e-cigarette use.

All analyses were conducted on weighted data using STATA version 17 (StataCorp, College Station, TX). We employed the "svy" command to account for the complex survey design utilized by the YRBS. A two-sided alpha of < 0.05 was used to determine statistical significance of the results.

## Results

Of the 12,767 participants, 49.5% were females, 13.4% were 18 years or older, 11.5% were African American, and 52.2% were White individuals. Approximately, 32.7% reported current e-cigarette use. Compared to individuals who reported no e-cigarette use, those who reported current e-cigarette use were more likely to be enrolled in 11^th^ grade or higher (55.5% vs. 43.2%), report current use of combustible cigarettes (15.9% vs 1.0%), smokeless tobacco (9.3% vs. 0.7%), cigar (14.4% vs. 0.9%), alcohol (64.8% vs. 12.2%) and marijuana (51.8% vs. 6.6%) (Table 1).

**Table 1:** Sociodemographic characteristics of study populations: no tobacco use vs. current e-cigarette use, 2019 youth risk behavior survey

**Sociodemographic Characteristics**	**Total** *N* = 12,767 (Weighted %)	**No e-cigarette use** *N* = 8,658 (Weighted % = 67.3%)	**Current e-cigarette use** *N* = 4,109 (Weighted % = 32.7%)
**Age**			
12–14	1,670 (12.4)	1,234 (13.7)	436 (10.0)
15	3,257 (25.0)	2,384 (27.4)	873 (25.6)
16	3,389 (25.6)	2,243 (25.1)	1,146 (23.9)
17	2,900 (23.6)	1,869 (22.3)	1,031 (22.3)
≥ 18	1,491 (13.4)	886 (11.4)	605 (18.2)
**Sex**			
Female	6,464 (49.5)	4,384 (49.0)	2,080 (50.7)
Male	6,183 (50.5)	4,208 (51.0)	1,975 (49.3)
**Race**			
AI/AN/PH/HI	192 (1.0)	108 (0.8)	84 (1.3)
Asian	585 (5.2)	504 (6.7)	81 (2.0)
African American	1,836 (11.5)	1,496 (13.8)	340 (6.9)
White	6,377 (52.2)	3,962 (48.0)	2,415 (60.8)
Hispanic	945 (9.2)	688 (9.9)	257 (7.8)
Multi-Racial	2,464 (20.9)	1,653 (20.8)	811 (22.1)
**Grade**			
9	3,412 (26.8)	2,554 (29.9)	858 (20.6)
10	3,483 (25.7)	2,404 (26.5)	1,079 (24.0
11	3,072 (24.1)	1,900 (22.9)	1,082 (26.5)
12	2,672 (23.4)	1,637 (20.7)	1,035 (29.0)
**Sexual Orientation**			
Heterosexual	10,217 (84.7)	6,932 (84.5)	6,531 (85.2)
Gay or Lesbian	330 (2.4)	219 (2.4)	203 (2.4)
Bisexual	1,068 (8.6)	697 (8.3)	635 (9.1)
Not sure	537 (4.3)	405 (4.8)	380 (3.3)
**Body Mass Index**			
Underweight	376 (2.7)	284 (3.0)	92 (2.1)
Normal weight	7,540 (58.2)	5,082 (57.7)	2,458 (59.3)
Overweight	1,804 (14.3)	1,191 (14.0)	613 (15.1)
Obese	3,047 (24.7)	2,101 (25.3)	946 (23.5)
**Current combustible cigarette use**			
No	10,970 (94.2)	7,874 (99.0)	3,096 (84.1)
Yes	667 (5.8)	82 (1.0)	585 (15.9)
**Current smokeless tobacco use**			
No	11,682 (96.5)	8,215 (99.3)	3,467 (90.7)
Yes	447 (3.5)	66 (0.7)	381 (9.3)
**Current cigar use**			
No	11,472 (94.7)	8,189 (99.1)	3,283 (85.6)
Yes	642 (5.3)	87 (0.9)	555 (14.4)
**Current alcohol use**			
No	8,537 (71.2)	7,221 (87.8)	1,316 (35.2)
Yes	3,406 (28.8)	1,035 (12.2)	2,371 (64.8)
**Current marijuana use**			
No	9,812 (78.7)	7,887 (93.4)	1,925 (48.2)
Yes	2,702 (21.3)	634 (6.6)	2,068 (51.8)

AI/AN/NH/PI American Indian/ Alaskan Native/Native Hawaiian/Pacific Islander

### Psychosocial stressors and current e-cigarette use

The weighted prevalence of current e-cigarette use was higher among individuals who experienced psychosocial stressors than those who did not: bullying (43.9% vs. 29.0%), sexual assault (58.5% vs. 31.4%), safety-related school absences (48.0% vs. 31.4%), depressive symptoms (43.5% vs. 26.5%), suicidal ideation (47.6% vs. 29.3%), physical altercations (51.1% vs. 28.3%), and weapon threats (56.4% vs. 30.9%) (Table 2).

**Table 2:** Weighted prevalence of psychosocial stressors and current e-cigarette use, 2019 youth risk behavior survey

**Psychosocial Stressors**	Weighted Prevalence of Psychosocial Stressors % (95% CI)	**Weighted Prevalence of** **Current E-cigarette Use % (95% CI)**
**Bullying**		
No (N = 9,426)	75.2 (73.7–76.6)	29.0 (26.7–31.4)
Yes (N = 3,187)	24.8 (23.5–26.3)	43.9 (41.3–46.6)
**Sexual Assault**		
No (N = 9,804)	93.0 (92.1–93.7)	31.4 (29.2–33.6)
Yes (N = 785)	7.0 (6.3–7.9)	58.5 (51.6–65.0)
**Safety-Related Absence from School**		
No (N = 11,644)	92.3 (91.2–93.3)	31.4 (29.4–33.6)
Yes (N = 1,063)	7.7 (6.7–8.8)	48.0 (43.1–52.9)
**Depressive Symptoms**		
No (N = 7,981)	63.4 (61.8–65.0)	26.5 (24.6–28.4)
Yes (N = 4,610)	36.6 (35.0–38.3)	43.5 (40.8–46.2)
**Suicidal Ideation**		
No (N = 10,162)	81.5 (80.2–82.7)	29.3 (27.2–31.4)
Yes (N = 2,437)	18.5 (17.3–19.8)	47.6 (44.6–50.6)
**Physical Altercations**		
No (N = 7,676)	77.0 (74.8–79.0)	28.3 (26.3–30.5)
Yes (N = 2,453)	23.0 (21.0–25.2)	51.1 (47.8–54.4)
**Weapon Threats**		
No (N = 11,756)	93.2 (92.3–94.1)	30.9 (28.9–33.0)
Yes (N = 900)	6.8 (6.0–7.7)	56.4 (52.2–60.5)
**Burden Score Categories**		
0 (N = 5,278)	41.4 (39.4–43.4)	20.5 (18.5–22.6)
1 (N = 3,205)	25.7 (24.4–27.0)	32.8 (30.0–35.6)
2 (N = 2,061)	16.1 (15.3–16.9)	41.4 (37.8–45.0)
3 (N = 1,280)	9.5 (8.9–10.1)	49.6 (44.5–54.7)
4,5,6, or 7 (N = 940)	7.3 (6.5–8.3)	60.9 (56.0–65.6)

In the multivariable-adjusted logistic regression analysis, participants who reported experiencing psychosocial stressors were significantly more likely to report current e-cigarette use than those who did not report the respective psychosocial stressor: bullying (OR [Odds ratio], 1.67; 95% CI [Confidence Interval]:1.37–2.03), sexual assault (OR, 1.70; 95% CI: 1.15–2.53), depressive symptoms (OR, 1.55; 95% CI: 1.31–1.84), suicidal ideation (OR, 1.40; 95% CI: 1.13–1.74), physical altercations (OR, 1.59; 95% CI:1.35–1.88), and weapon threats (OR, 1.75; 95% CI:1.32–2.32) (Table 3). When restricting our sample to individuals who did not report other tobacco product use, the association between psychosocial stressors and current sole e-cigarette use remained significant (Supplementary Table 2).

**Table 3:** Association between individual psychosocial stressors and current e-cigarette use, 2019 youth risk behavior survey

Psychosocial Stressors	Model 1 aOR (95% CI)	Model 2 aOR (95% CI)	Model 3 aOR (95% CI)
**Bullying**			
No	Reference	Reference	Reference
Yes	**1.97 (1.71–2.28)**	**1.75 (1.49–2.04)**	**1.67 (1.37–2.03)**
**Sexual Assault**			
No	Reference	Reference	Reference
Yes	**3.02 (2.20–4.15)**	**2.36 (1.68–3.32)**	**1.70 (1.15–2.53)**
**Safety-Related Absence from School**			
No	Reference	Reference	Reference
Yes	**2.08 (1.61–2.69)**	**1.73 (1.31–2.30)**	1.47 (0.99–2.18)
**Depressive Symptoms**			
No	Reference	Reference	Reference
Yes	**2.31 (2.04–2.60)**	**2.06 (1.83–2.31)**	**1.55 (1.31–1.84)**
**Suicidal ideation**			
No	Reference	Reference	Reference
Yes	**2.46 (2.14–2.84)**	**2.07 (1.79–2.40)**	**1.40 (1.13–1.74)**
**Physical Altercations**			
No	Reference	Reference	Reference
Yes	**3.24 (2.83–3.71)**	**2.47 (2.11–2.88)**	**1.59 (1.35–1.88)**
**Weapon Threats**			
No	Reference	Reference	Reference
Yes	**3.07 (2.52–3.74)**	**2.17 (1.73–2.72)**	**1.75 (1.32–2.32)**

Model 1: Adjusted for age, sex, race and ethnicity, sexual orientation, and body mass index

Model 2: Model 1 + current combustible cigarette, cigar, and smokeless tobacco use

Model 3: Model 2 + current alcohol and marijuana use

**Fig. 1:**
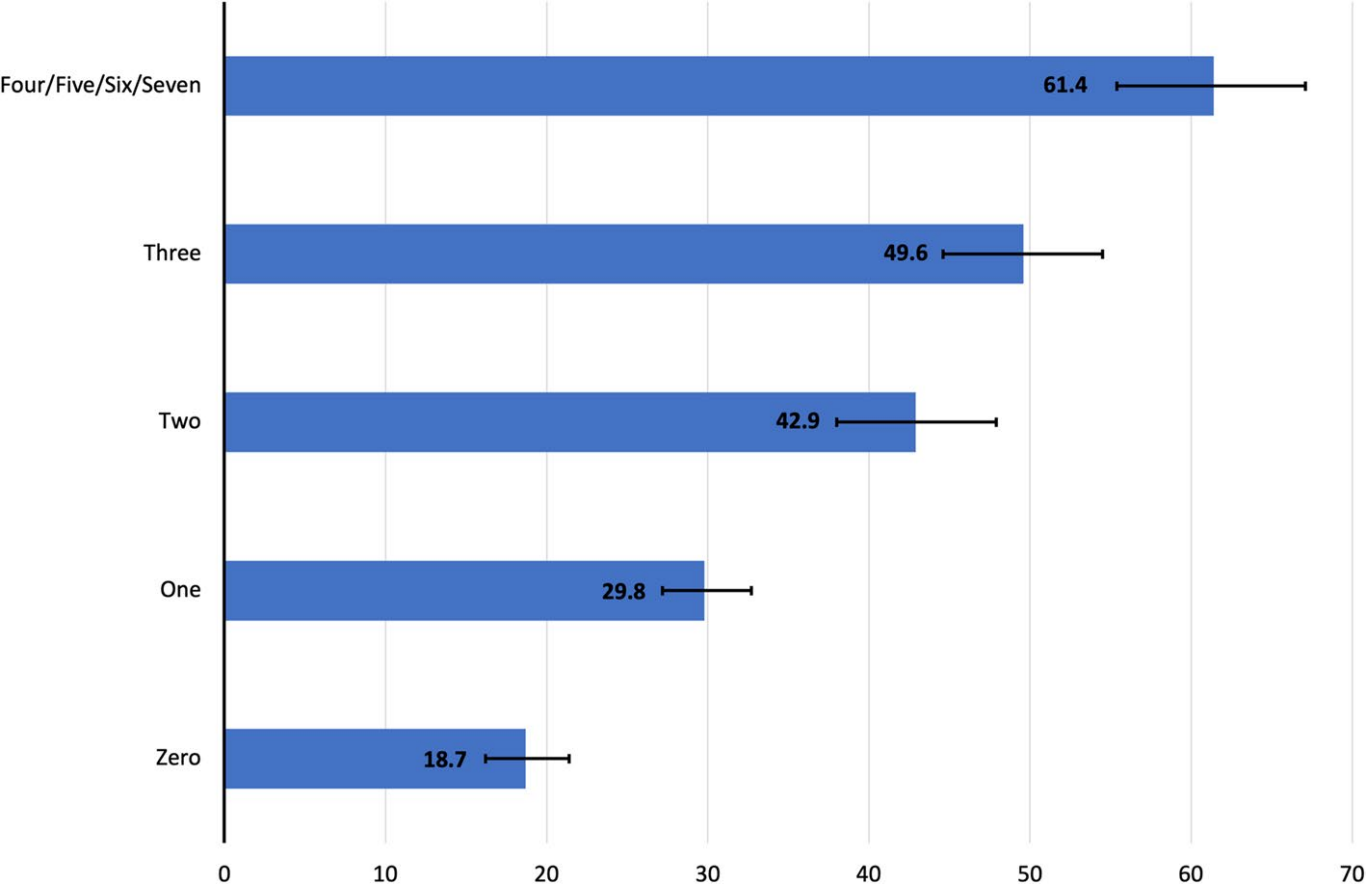
Weighted Prevalence of Current E-cigarette Use by Psychosocial Stressor Burden Score

The prevalence of current e-cigarette use increased with increasing psychosocial stressor burden scores (Fig. 1 and Table 2) and was comparable between females and males (Supplementary Fig. 1a and b). Compared to individuals with a psychosocial stressor burden score of zero, those with higher burden scores had significantly higher and graded odds of current e-cigarette use: one (OR, 1.43; 95% CI: 1.20–1.71), two (OR, 1.81; 95% CI: 1.39–2.37), and three (OR, 2.31; 95% CI: 1.73–3.07) and four to seven (OR, 2.73; 95% CI: 2.06–3.64) (Table 4). In sensitivity analysis modelling the stressor burden score as continuous, there remained a significant association between psychosocial stressor burden score and e-cigarette use (OR, 1.28; 95% CI: 1.20–1.35) (Table 4).

**Table 4:** Association between psychosocial stressor burden scores and current e-cigarette use, 2019 youth risk behavior survey

Burden scores	Model 1 aOR (95% CI)	Model 2 aOR (95% CI)	Model 3 aOR (95% CI)
Burden score	**1.60 (1.52–1.69)**^a^	**1.47 (1.40–1.55)**^a^	**1.28 (1.20–1.35)**^a^
0	Reference	Reference	Reference
1	**2.06 (1.77–2.39)**	**1.90 (1.61–2.25)**	**1.43 (1.20–1.71)**
2	**3.08 (2.59–3.67)**	**2.61 (2.17–3.14)**	**1.81 (1.39–2.37)**
3	**4.52 (3.56–5.74)**	**3.52 (2.83–4.38)**	**2.31 (1.73–3.07)**
4,5,6, or 7	**7.74 (5.82–10.29)**	**5.29 (3.98–7.05)**	**2.73 (2.06–3.64)**

Model 1: Adjusted for age, sex, race and ethnicity, sexual orientation, and body mass index

Model 2: Model 1 + current combustible cigarette, cigar, and smokeless tobacco use

Model 3: Model 2 + current alcohol and marijuana use

*aOR* Adjusted odds ratio, *CI* Confidence interval

^a^Modelled as a continuous variable

*p*-value for linear trend for all models < 0.001

There was a significant association between the individual psychosocial stressors and current combustible cigarette use, with the strength of the association similar to that between the stressors and e-cigarette use (Supplementary Table 3).

## Discussion

Utilizing the large and nationally representative data of U.S. high school students, our study highlighted a significant association between psychosocial stressor and current e-cigarette use, with higher e-cigarette use prevalence among individuals who reported experiencing stressors compared to those who did not. Additionally, the prevalence and odds of e-cigarette use increased in a graded manner with increasing psychosocial stressor burden scores. Also, in the supplemental analysis, we demonstrate significant associations between individual psychosocial stressors and combustible cigarette use, with the strength of the association comparable to that of e-cigarettes. These findings suggest that psychosocial stressors may influence adolescent e-cigarette and combustible cigarette use, further highlighting that stress reduction among adolescents could be a means to potentially reduce tobacco use in this population.

Previous research has shown a positive association between some psychosocial stressors such as bullying, family conflict, academic pressure, and e-cigarette use among adolescents [25–28]. For example, one study demonstrated that adolescents who experienced bullying had 1.5 to 2 times higher odds of e-cigarette use than those who did not encounter bullying [28]. Our findings are consistent with this observation, indicating a 1.67 times higher likelihood of e-cigarette use among adolescents who reported that they had been bullied. Moreover, our study elucidates the significant association between stressors and e-cigarette use, showing that other forms of stressors, such as sexual assault, depressive symptoms, physical altercations, and weapon threats, are associated with 1.40 to 1.75 times significantly higher odds of e-cigarette use compared to adolescents without such experiences. Importantly, we also demonstrate a dose–response association, showing that participants with higher burden scores had a higher prevalence and 1.43 to 2.73 times higher odds of e-cigarette use compared to those with burden scores of zero. Furthermore, our study found that the strength of the association between psychosocial stressors and e-cigarette use is comparable to that of combustible cigarette use, which has been also shown in previous studies to be significantly associated with various psychosocial stressors [29, 30]. These findings highlight the intricate interactions between psychosocial stressors and adolescent tobacco use.

Adolescents frequently encounter stressors such as peer pressure, academic challenges, and family issues, which may increase their likelihood of using e-cigarettes [25–28]. In this study, participants with higher burden scores, indicating greater exposure to psychosocial stressors, had a higher prevalence and odds of e-cigarette use than those without stressor experiences. These findings suggest that the accumulation of multiple stressors may exacerbate the association between stressors and e-cigarette use among adolescents. It is crucial to address adolescent e-cigarette use as they may turn to these products to cope with stress, which may indicate maladaptive coping mechanisms. The convenience and discreetness of e-cigarettes make them an attractive option for youth dealing with various forms of stress [31, 32]. Additionally, the lack of stricter enforcement of age-related sales restrictions making e-cigarettes easily accessible to youth, coupled with peer pressure, and the appeal of various flavors may contribute to adolescent use e-cigarettes to cope with stress [25, 33–36]. Targeted advertising by e-cigarette companies, especially during vulnerable periods such as times of stress, has been found to increase adolescent use [35–37]. This, compounded with some evidence suggesting that e-cigarettes may serve as a gateway to combustible cigarette use, further emphasizes the need to develop targeted intervention strategies to promote healthier coping mechanisms and prevent youth tobacco use during times of stress.

Our findings emphasize the critical need to address the intricate associations between various psychosocial stressors and high-risk behaviors such as e-cigarette use among adolescents. A comprehensive approach that includes mental health support, stringent control of e-cigarette and other tobacco product access, and marketing regulations is warranted to mitigate adolescent tobacco use. Mental health resources, including counseling and stress management programs, should be integrated into educational and community settings to facilitate adaptive coping strategies among adolescents. Concurrently, more stringent regulations may encompass enhanced enforcement of the minimum purchasing age, increased penalties for noncompliant retailers, expanded bans on flavored e-cigarettes, and tightened restrictions on youth-targeted marketing. Targeted educational campaigns, such as the Real Cost Campaign, can be beneficial in reducing adolescent e-cigarette use [38]. The Real Cost Campaign is a public health initiative that aims to prevent and reduce tobacco use among adolescents by highlighting the negative consequences of tobacco use. Thus, in addition to stricter enforcement of existing policies and educational campaigns, the findings of our study suggest that helping adolescents develop and adopt healthy ways of coping with stress may be instrumental in reducing adolescent tobacco use. This comprehensive approach can promote healthier behaviors, reduce e-cigarette use, and improve well-being within this vulnerable population.

### Limitations

While this study benefits from a large, nationally representative dataset, it is essential to interpret the findings cautiously as there are some limitations. Our study uses data that is representative of high school students in the US, and hence older adolescents, and may not be generalizable to younger adolescents or youth who are not enrolled in schools. Self-reported data raises concerns of recall and misclassification bias, which cannot be excluded. Additionally, certain factors that could potentially impact the association between stressors and e-cigarette use, such as peer pressure, home characteristics, parental support, and school type, were not assessed in the YRBS and hence were not adjusted for in our analysis. There is therefore the potential for residual confounding of the associations assessed. Furthermore, the study's observational and cross-sectional design precludes any causal inference, and thus it is unclear whether stressors are a cause or consequence of e-cigarette use.

## Conclusion

Our study demonstrates a significant association of psychosocial stressors such as bullying, sexual assault, and safety-related absence from school with adolescent e-cigarette and combustible cigarette use, highlighting the need for a comprehensive approach that includes mental health support and school health programs in addition to stringent enforcement of access and marketing regulations as means of reducing youth tobacco use. Implementing these comprehensive measures may promote healthier behaviors and improve overall well-being among this vulnerable population.

## Supplementary Information

**Additional file 1: Supplementary Table 1.** Questions Used to Assess Psychosocial Stressors, 2019 Youth Risk Behavior Survey.

**Additional file 2: Supplementary Table 2.** Association between Individual Psychosocial Stressors and Sole E-Cigarette Use, 2019 Youth Risk Behavior Survey.

**Additional file 3: Supplementary Table 3.** Association between Individual Psychosocial Stressors and Current Combustible Cigarette Use, 2019 Youth Risk Behavior Survey.

**Additional file 4: Supplementary Figure 1a.** Weighted Prevalence of Current E-cigarette Use by Psychosocial Stressor Burden Score for Males. **Figure 1b.** Weighted Prevalence of Current E-cigarette Use by Psychosocial Stressor Burden Score for Females.

## Data Availability

The publicly available data can be found here: https://www.cdc.gov/healthyyouth/data/yrbs/data.htm.

## Abbreviations

U.S.: United States
OR: Odds Ratio
NIH: National Institutes of Health
FDA: Food and Drug Administration
