# Supplementary material for: Psychosocial stressors and current e-cigarette use in the youth risk behavior survey
Source: BMC Public Health. 2023 Jun 6;23:1080. doi: 10.1186/s12889-023-16031-w (PMC10242777; doi:10.1186/s12889-023-16031-w)
Supplement: Supplementary file 1 — Additional file 1: Supplementary Table 1. Questions Used to Assess Psychosocial Stressors, 2019 Youth Risk Behavior Survey. [file 12889_2023_16031_MOESM1_ESM.docx]

Supplementary Table 1: ﻿ Questions Used to Assess Psychosocial Stressors, 2019 Youth Risk Behavior Survey

| **Psychosocial stressors** | **Assessment questions** |
| --- | --- |
| **Bullying** | “During the past 12 months, have you ever been bullied on school property?”  “During the past 12 months, have you ever been electronically bullied?” |
| **Sexual Assault** | “Have you ever been physically forced to have sexual intercourse when you did not want to? |
| **Safety-related absences from school** | “During the past 30 days, on how many days did you not go to school because you felt you would be unsafe at school or on your way to or from school?” |
| **Depressive Symptoms** | During the past 12 months, did you ever feel so sad or hopeless almost every day for **two weeks or more in a row** that you stopped doing some usual activities? |
| **Suicidal Ideation** | During the past 12 months, did you ever **seriously** consider attempting suicide? |
| **Physical Altercations** | “During the past 12 months, how many times were you in a physical fight?”  “During the past 12 months, how many times were you in a physical fight on school property” |
| **Weapon Threats** | “During the past 12 months, how many times has someone threatened or injured you with a weapon such as a gun, knife, or club on school property?” |
| *Participants who answered "yes" to the questions were classified as having experienced the stressor.* | |
