# Supplementary material for: Psychosocial stressors and current e-cigarette use in the youth risk behavior survey
Source: BMC Public Health. 2023 Jun 6;23:1080. doi: 10.1186/s12889-023-16031-w (PMC10242777; doi:10.1186/s12889-023-16031-w)
Supplement: Supplementary file 2 — Additional file 2: Supplementary Table 2. Association between Individual Psychosocial Stressors and Sole E-Cigarette Use, 2019 Youth Risk Behavior Survey. [file 12889_2023_16031_MOESM2_ESM.docx]

Supplementary Table 2: ﻿ Association between Individual Psychosocial Stressors and Sole E-Cigarette Use, 2019 Youth Risk Behavior Survey

| **Psychosocial Stressors** | **Model 1**  **OR (95% CI)** | **Model 2**  **OR (95% CI)** |
| --- | --- | --- |
| **Bullying** |  |  |
| No | Reference | Reference |
| Ye | **1.76 (1.49-2.06)** | **1.67 (1.36-2.05)** |
| **Sexual Assault** |  |  |
| No | Reference | Reference |
| Yes | **2.40 (1.72-3.36)** | **1.69 (1.13-2.54)** |
| **Safety-Related Absence from School** |  |  |
| No | Reference | Reference |
| Yes | **1.83 (1.37-2.46)** | **1.60 (1.07-2.39)** |
| **Depressive Symptoms** |  |  |
| No | Reference | Reference |
| Yes | **2.07 (1.84-2.32)** | **1.56 (1.30-1.87)** |
| **Suicidal Ideation** |  |  |
| No | Reference | Reference |
| Yes | **2.10 (1.83-2.42)** | **1.37 (1.10-1.71)** |
| **Physical Altercations** |  |  |
| No | Reference | Reference |
| Yes | **2.48 (2.10-2.92)** | **1.57 (1.29-1.93)** |
| **Weapon Threats** |  |  |
| No | Reference | Reference |
| Yes | **2.16 (1.72-2.72)** | **1.70 (1.32-2.20)** |
| aOR, Adjusted odds ratio; CI, Confidence interval  Analysis was restricted to participants who did not use combustible cigarettes, smokeless tobacco, or cigars.  Model 1: Adjusted for age, sex, race and ethnicity, sexual orientation, and body mass index.  Model 2: Model 1 + current alcohol and marijuana use | | |
