# Supplementary material for: Psychosocial stressors and current e-cigarette use in the youth risk behavior survey
Source: BMC Public Health. 2023 Jun 6;23:1080. doi: 10.1186/s12889-023-16031-w (PMC10242777; doi:10.1186/s12889-023-16031-w)
Supplement: Supplementary file 3 — Additional file 3: Supplementary Table 3. Association between Individual Psychosocial Stressors and Current Combustible Cigarette Use, 2019 Youth Risk Behavior Survey. [file 12889_2023_16031_MOESM3_ESM.docx]

Supplementary Table 3: ﻿ Association between Individual Psychosocial Stressors and Current Combustible Cigarette Use, 2019 Youth Risk Behavior Survey

| Psychosocial Stressors | **Model 1**  **OR (95% CI)** | **Model 2**  **OR (95% CI)** | **Model 3**  **OR (95% CI)** |
| --- | --- | --- | --- |
| **Bullying** |  |  |  |
| No | Reference | Reference | Reference |
| Yes | **2.00 (1.48-2.70)** | 1.34 (0.94-1.90) | 1.34 (0.93-1.95) |
| **Sexual Assault** |  |  |  |
| No | Reference | Reference | Reference |
| Yes | **4.32 (3.10-6.01)** | **1.94 (1.27-2.97)** | **1.68 (1.05-2.69)** |
| **Safety-Related Absence from School** |  |  |  |
| No | Reference | Reference | Reference |
| Yes | **2.39 (1.71-3.34)** | **1.50 (1.09-2.06)** | **1.52 (1.02-2.27)** |
| **Depressive Symptoms** |  |  |  |
| No | Reference | Reference | Reference |
| Yes | **2.44 (1.99-3.00)** | **1.65 (1.25-2.18)** | **1.41 (1.04-1.90)** |
| **Suicidal Ideation** |  |  |  |
| No | Reference | Reference | Reference |
| Yes | **3.31 (2.57-4.26)** | **2.12 (1.58-2.85)** | **1.71 (1.25-2.35)** |
| **Physical Altercations** |  |  |  |
| No | Reference | Reference | Reference |
| Yes | **4.18 (3.14-5.58)** | **1.82 (1.29-2.58)** | **1.46 (1.01-2.13)** |
| **Weapon Threats** |  |  |  |
| No | Reference | Reference | Reference |
| Yes | **4.42 (3.26-5.98)** | **2.06 (1.38-3.07)** | 1.63 (0.97-2.75) |
| aOR, Adjusted odds ratio; CI, Confidence interval  Model 1: Adjusted for age, sex, race and ethnicity, sexual orientation, and body mass index.  Model 2: Model 1 + current e-cigarette, cigar, and smokeless tobacco use  Model 3: Model 2 + current alcohol and marijuana use | | | |
