# Supplementary material for: Psychosocial stressors and current e-cigarette use in the youth risk behavior survey
Source: BMC Public Health. 2023 Jun 6;23:1080. doi: 10.1186/s12889-023-16031-w (PMC10242777; doi:10.1186/s12889-023-16031-w)
Supplement: Supplementary file 4 — Additional file 4: Supplementary Figure 1a. Weighted Prevalence of Current E-cigarette Use by Psychosocial Stressor Burden Score for Males. Figure 1b. Weighted Prevalence of Current E-cigarette Use by Psychosocial Stressor Burden Score for Females. [file 12889_2023_16031_MOESM4_ESM.zip › Supplementary Figure 1a.pdf]

Supplementary Figure 1a: Weighted Prevalence of Current E-cigarette Use by Psychosocial Stressor Burden Score for **Males**

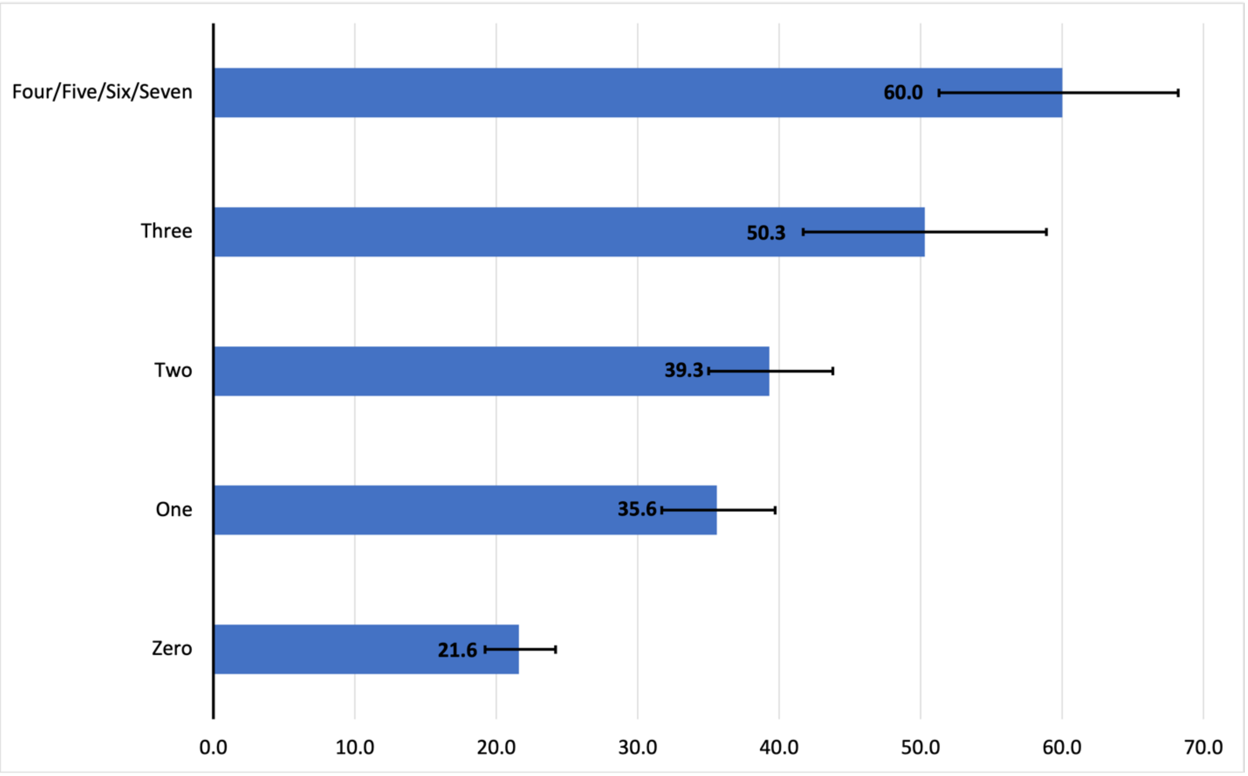

The figure shows the weighted prevalence of current e-cigarette use across psychosocial stressor burden scores (0,1,2,3,4-7) for males. The point estimates are represented by the upper limit of the bar chart, and the 95% CI are represented by the upper and lower horizontal bars.
