# Supplementary material for: Psychosocial stressors and current e-cigarette use in the youth risk behavior survey
Source: BMC Public Health. 2023 Jun 6;23:1080. doi: 10.1186/s12889-023-16031-w (PMC10242777; doi:10.1186/s12889-023-16031-w)
Supplement: Supplementary file 4 — Additional file 4: Supplementary Figure 1a. Weighted Prevalence of Current E-cigarette Use by Psychosocial Stressor Burden Score for Males. Figure 1b. Weighted Prevalence of Current E-cigarette Use by Psychosocial Stressor Burden Score for Females. [file 12889_2023_16031_MOESM4_ESM.zip › Supplementary Figure 1b.pdf]

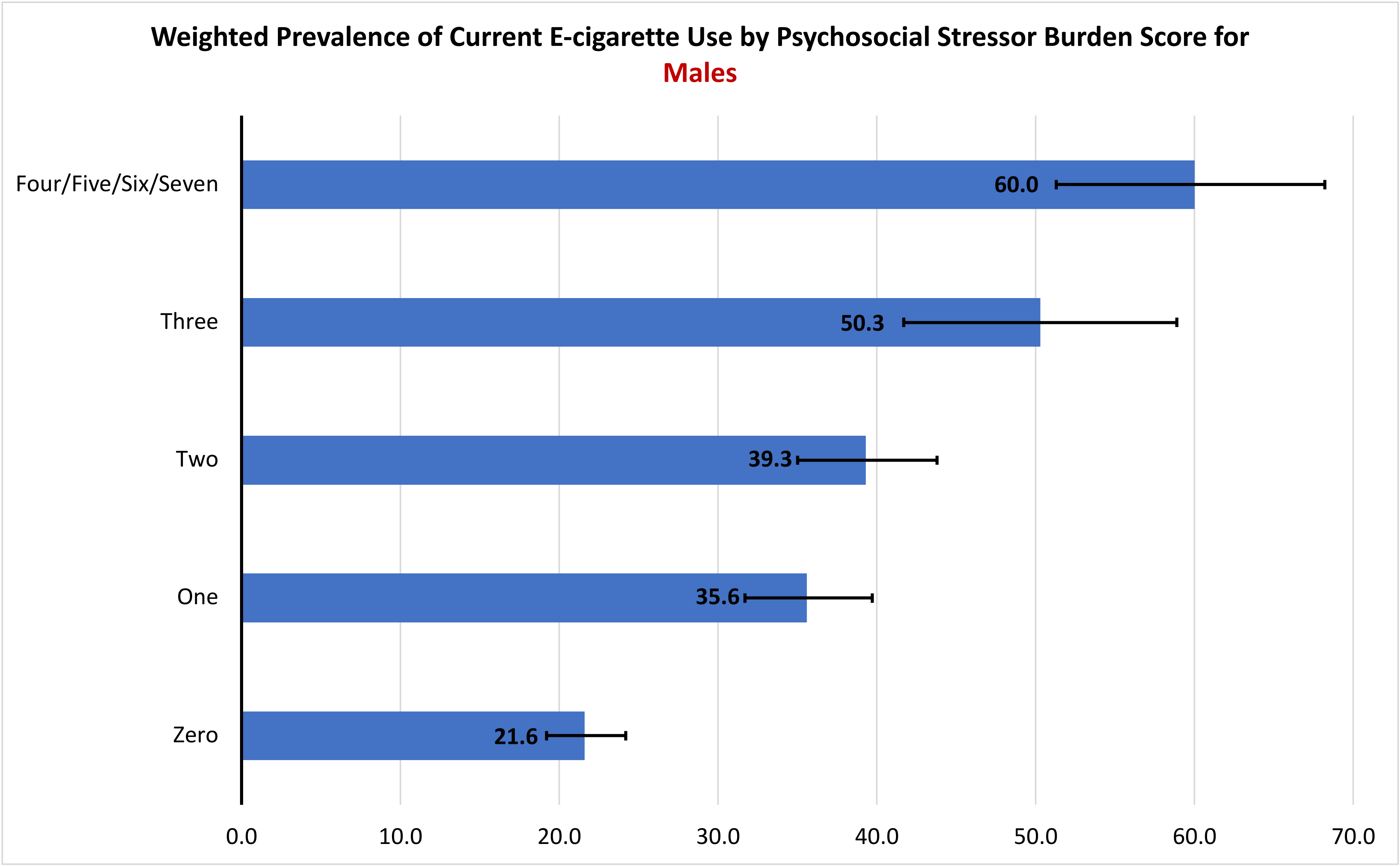
Weighted Prevalence of Current E-cigarette Use by Psychosocial Stressor Burden Score for Males
Four/Five/Six/Seven
Three
Two
One
Zero
60.0
50.3
39.3
35.6
21.6
0.0
10.0
20.0
30.0
40.0
50.0
60.0
70.0
